# States of Resistance: nosocomial and environmental approaches to antimicrobial resistance in Lebanon

**DOI:** 10.1007/s40656-024-00624-8

**Published:** 2024-08-01

**Authors:** Louis-Patrick Haraoui, Anthony Rizk, Hannah Landecker

**Affiliations:** 1 Department of Microbiology and Infectious Diseases, Faculty of Medicine and Health Sciences, Université de Sherbrooke, Sherbrooke, QC Canada; 2 Centre de Recherche Charles-Le Moyne, CISSS Montérégie-Centre, Greenfield Park, QC Canada; 3 Department of Anthropology and Sociology, Geneva Graduate Institute (IHEID), Geneva, Switzerland; 4 Department of Sociology, Institute for Society and Genetics, 264 Haines Hall, 375 Portola Plaza, Los Angeles, CA 90095 USA

**Keywords:** Antimicrobial resistance, Lebanon, Armed conflict, Acinetobacter baumannii, Hospital hygiene, One Health

## Abstract

Drawing on institutional historical records, interviews and student theses, this article charts the intersection of hospital acquired illness, the emergence of antimicrobial resistance (AMR), environments of armed conflict, and larger questions of social governance in the specific case of the American University of Beirut Medical Center (AUBMC) in Lebanon. Taking a methodological cue from approaches in contemporary scientific work that understand non-clinical settings as a fundamental aspect of the history and development of AMR, we treat the hospital as not just nested in a set of social and environmental contexts, but frequently housing within itself elements of social and environmental history. AMR in Lebanon differs in important ways from the settings in which global protocols for infection control or rubrics for risk factor identification for resistant nosocomial outbreaks were originally generated. While such differences are all too often depicted as failures of low and middle-income countries (LMIC) to maintain universal standards, the historical question before us is quite the reverse: how have the putatively universal rubrics of AMR and hospital infection control failed to take account of social and environmental conditions that clearly matter deeply in the evolution and spread of resistance? Focusing on conditions of war as an organized chaos in which social, environmental and clinical factors shift dramatically, on the social and political topography of patient transfer, and on a missing “meso” level of AMR surveillance between the local and global settings, we show how a multisectoral One Health approach to AMR could be enriched by an answering multisectoral methodology in history, particularly one that unsettles a canonical focus on the story of AMR in the Euro-American context.

## Introduction

In the annals of history of antibiotic resistant organisms, hospitals loom large as the places in which these microbes were first noticed, most intensively surveilled, and indeed initially conceptualized as the likely origin of many resistant strains in the first place (Gradmann, 2018). “Hospital *Staphylococcus*” was the colloquial moniker given to the first great epidemic wave of penicillin-resistant staph to sweep the world after the introduction of antibiotics in World War II (Shooter, 1981). The history of the development of hospital infection control as a distinct subspecialty in nursing, clinical practice, and research is inextricably entwined with the emergence of the resistant hospital-acquired infection (Rafferty et al., 2021). The early years of research into antimicrobial resistance (AMR) and efforts to control it were therefore almost exclusively focused on medical care settings in which antibiotics were being used as therapies.

In recent years however, a growing awareness of the importance of non-clinical settings to the evolution and spread of AMR has emerged, including the use of antibiotics in agriculture, the circulation of antimicrobial substances, resistant organisms and genetic resistance determinants in waste water, and the role of natural environments in which microbial genes with potential to confer resistance have ancient and long-evolved ecological functions (Castillo-Ramírez et al., 2021). A “One Health” perspective on AMR has advocated for a multisectoral integration of research on agricultural, aquatic, and wildlife settings with the question of resistance in the clinical realm, on the grounds that the world is interconnected, and thus the research must also be (White & Hughes, 2019). The hospital or the clinic is not an island unto itself.

One Health has not yet been met with a commensurate “One History” approach to AMR in the historical sciences. Of course this does not mean that there should be a single history, rather the opposite: in the spirit of the One Health concept *and* its critiques, environmental, social, and natural histories should crowd and complicate the frame that has to date been constructed by classical approaches in the history of “Western” science and medicine dominated by a focus on clinical matters in British, European or North American people and places (Bud, 2007; Elshakry, 2010; Hinchliffe, 2015; Podolsky, 2015; Santesmases, 2017). A demonstrated weak point of One World One Health (OWOH) aspirations is a focus on singularity and commonality, and the assumption that scientific solutions for the world will “spread outward, from center to periphery, in a uniform predictable pattern”; these assumptions undermine the efficacy of interventions when they meet the actual multiplicity and variegation of the many different worlds constituting a global reality (Yates-Doerr, 2015, p. 111). These assumptions about diffusion may indeed originate in part from the historiography of microbiology itself, which, as is highlighted by this special issue, tends to focus on a few key European moments of origin and approach everything as a genealogical descendent of them.

Hospital hygiene is an excellent exemplar of just such a “mobile scientific sovereignty” that assumes the world to be a uniform surface to be sterilized (Pandolfi, 2009). The hospital is a particularly placeless place—a “simple backdrop or static scenery” in which humans enact relationships of control vis-a-via microbes, rather than a culturally and historically particular place shaped and transformed by the evolution of microbes within it (Brives, 2021, p. 261). By providing the many microbial stories from the many worlds that are hospitals, and thinking about how they interconnect, the historical sciences and ethnographic methods have the capacity to improve the understanding of the socio-cultural environments determining the course of AMR, which in the One Health framework are relatively underemphasized due to its origins in veterinary and environmental science.

Increased attention “to how social arrangements are entangled with changing material conditions, producing vulnerabilities for particular people and animals in particular places and times” is much needed to extend the classic notion of local or situated human biology to a framework for understanding AMR as a situated microbiology (Helliwell et al., 2021, p. 1363; Koch, 2011; Niewöhner & Lock, 2018). Excellent work on the use of antibiotics in agriculture and a small but growing literature on the history of concerns about antibiotics in environmental microbiology begin to point the way toward understanding the environment beyond the clinic as integral to histories of AMR, yet this is not just a question of adding extra-clinical topics or animal-human relations or other places to the mix (Kirchhelle, 2018; Rasmussen, 2022). Rather, it is a methodological question of how to complement multisectoral research in the present with an equivalently multisectoral historical approach, resituating the clinic in the contexts that matter to it and vice-versa (Brives, 2022).

Here we take up the question of the antibiotic resistant nosocomial infection and efforts to control it in the history of one particular institution, the American University of Beirut Medical Center (AUBMC). We demonstrate that theories of hospital infection and their subsequent historiography, generated almost exclusively from British and American institutions, are confirmed and echoed to a certain extent by efforts to instantiate these apparently universal principles of infection control and risk factor research; and yet at the same time are profoundly challenged by the specific local events and conditions of the physical and social environment. Taking our cue from approaches in contemporary scientific work that understand non-clinical settings as a fundamental aspect of the history and development of AMR, we treat the hospital as not just nested within a set of social and environmental contexts, but frequently housing within itself elements of social and environmental history. In other words, this is not necessarily a content-context picture of a hospital in a context, but that the context often walks in the door and becomes an intrinsic part of the microbiological history of a place. Drawing on the hospital’s series of Annual Reports, student theses, and interviews with researchers and clinicians, we chart the intersection of hospital-acquired illness, the emergence of AMR, and larger questions of environmental and social governance in the specific case of the AUBMC.^1^

In what follows, we briefly review the standard historiography of hospital infection control, before turning to the question of how it unfolds at AUBMC, which has its own long regionally situated history. The institution dates to 1862, when United States missionaries in the Ottoman Empire (part of which became present-day Lebanon in 1943) began the process of founding a college of higher learning that would include medical training. As detailed in Marwa Elshakry’s elegant analysis of the stakes implicit in missionary propagation of science in the Middle East, the Syrian Protestant College (SPC), chartered in the State of New York, was established in Beirut in 1866 (2007). The following year, the SPC inaugurated its “Medical Department,” later to become the School of Medicine, attracting and training local and regional elites throughout the Middle Eastern territories of the Ottoman Empire (Dewachi, 2017). The Syrian Protestant College was renamed the American University of Beirut in 1920 and started a period of expansion and modernization with the financial support of the Rockefeller Foundation, setting the stage for the American University of Beirut and other knowledge institutions like it to become a regionally connective force that guided post-mandate period decolonization across the Middle East (Schayegh, 2015a, 2015b). Part of this expansion was the establishment of the Department of Bacteriology, Pathology, Hygiene and Parasitology. Laboratory work previously done separately in each medical pavilion became centralized in 1925 and housed in the newly built Pathology Building, no longer in use today (Zaatari, 2015).

Run by a “skeleton full-time crew and few visiting faculty members” between 1925 and 1970, these laboratories attracted specimens from across the region as the reputation of the hospital grew. During shortages in World War II, the School of Medicine’s Bacteriology Department was relied on by the government to produce vaccines for plague, cholera, smallpox and typhoid-paratyphoid A and B. In 1970, 5 years before the onset of the Lebanese Civil War (1975–1990), an expanded medical center, the American University Hospital (AUH), was inaugurated, furnishing the Department of Bacteriology with “more spacious diagnostic facilities and modern technologies” (Zaatari, 2015).

The AUH played a critical role throughout the civil war years in providing medical care to the wounded and would become, itself, a key site of conflict between warring parties vying for control of the medical center and its resources (Mouro, 1999). AUH was re-branded as the American University of Beirut Medical Center (AUBMC) in 1999 and continues to hold this name today. The annual reports of the AUBMC, theses produced by students of the Department of Bacteriology, and interviews we rely on begin for the most part with the middle span of this history, from the mid-1950s onward.

The case of antimicrobial resistance in this place illustrates the specific trajectory of AMR as a research problem in a location that is in important ways different from the settings in which global protocols for infection control or rubrics for risk factor identification for resistant nosocomial outbreaks were originally generated. While such differences are all too often depicted as failures of low and middle-income countries (LMIC) to maintain universal standards and appropriate surveillance (Gradmann & Kirchhelle, 2023), the historical question before us is quite the reverse: *how have the putatively universal rubrics of AMR failed to take account of social and environmental conditions that clearly matter deeply in the evolution and spread of resistance*? Our work builds on the growing sense that for antibiotics and AMR, “one-size-fits-all policies, framed with the developed country context in mind” are inappropriate outside of those contexts (Kakkar et al., 2018), and a small but important literature that sees AMR not as a universal problem but as a highly differentiated one occurring around the world at different intersections of “colonial governance and humanitarianism” (Lopez et al., 2022, p. 1). Complementing these insights, we show that empirical work pursued in settings different from affluent Western nations may very well show up salient aspects of AMR as a social phenomenon, aspects that should be informative for everyone, not just inhabitants of low-income countries. Understanding hospital infection control as an effort to maintain control in a local environment fundamentally shaped by other social factors such as periods of political unrest, social or economic instability, and warfare is to firmly place the hospital in the world, and the world in the hospital.

## Hospital hygiene: the standard narrative

The history of hospital hygiene stretches far further back than that of AMR. Historians of medicine have charted a rough periodization for Europe and North America in which advocacy for improved sanitary standards and operative procedures from Florence Nightingale and Joseph Lister resulted in the development of wound dressing and cleaning rituals in the late nineteenth century, while practical developments such as gloves, surgical masks and isolation practices were introduced between 1890 and 1930, alongside new antiseptics (Rafferty et al., 2021; Schlich, 2013). It was a period in which “therapeutic modernity and meticulous hygiene were closely connected,” and the introduction of sulfonamides and then antibiotics beginning in the 1930s seemed at first a powerful extension of this connection (Gradmann, 2018, p. 2). It is not coincidental that the developer of the first sulfonamide drugs, Gerhard Domagk, simultaneously invented an impactful class of biocidal surface disinfectants called quaternary ammonium compounds (Landecker, 2019; Lesch, 2007). World War I and World War II brought research attention to the question of the bacterial flora of wounds over time, and the incidence of cross infection between wounds, particularly with hemolytic *Streptococcus* (Miles et al., 1940).

Thus, the problem of hospital infection, sharpened by world war, was a long-standing scene into which antibiotic therapy entered, yet this entry paradoxically reshaped hospital infection as a medical problem and a focus of research. As Drs. Williams, Blowers, Garrod and Shooter remarked rather acerbically in 1960 in their jointly authored volume *Hospital Infection*:

The past ten years of struggle with an epidemic antibiotic resistant strain of *Staphylococcus aureus* in hospitals around the world was far more disturbing than previous outbreaks of bacterial disease that occurred before an effective therapy for them was available. This reappearance of an old problem *has caused much greater disquiet than did its existence in the days when no treatment was available*. It has demanded and produced a very thorough re-investigation of the ways in which pathogenic organisms spread in hospitals. Not only have efforts been made in many hospitals to trace the migrations of individual strains of bacteria, but the efficacy of proceedings designed to prevent this have been re-examined, sometimes with highly disturbing results (Williams, 1960, p. 1). (Emphasis added)

Indeed, this worldwide staphylococcal pandemic of the 1950s *“*catalyzed the first systematic efforts to understand and control hospital infection in the United States” (Sharpe et al., 1998, p. 158). In 1959, the Centers for Disease Control (CDC) opened a *Staphylococcus* Surveillance Unit focused on hospitals. Hospital infection surveillance programs were added to the standards set by the Joint Commission on the Accreditation of Hospitals, quickly followed by requirements such as the isolation of infected patients, the provisioning of microbial scientific services such as phage typing and antibiotic susceptibility testing in strains found in patients, and infection control measures directed specifically at obstetrics and food services. Similar frameworks unfolded in Britain, where Kathryn Hillier has argued that staphylococcal infections in neonatal units during this epidemic were key in “transforming the role of the hospital bacteriologist from mere technician into infection-control expert,” as a new class of professionals was charged with responsibility for such measures (2006, p. 733).

In the 1970s, the CDC defined a nosocomial infection as one that develops in a patient after admission to a hospital when that infection was neither present nor in the incubating stage at the time of the patient’s admission, and established a National Nosocomial Infections Surveillance System (NNIS) (Garner et al., 1988). The 1970s and 1980s saw a rapid professionalization of roles such as infection control nursing, including the formation of professional societies for nurses, doctors, and researchers involved specifically in the task of infection control (Larson, 1997). This period also saw the rise of concern about methicillin resistant *Staphylococcus aureus* (MRSA) and a new focus on behavioral control around handwashing and isolation, as well as research focused on particular risk factors such as intrusive medical device use in hospital outbreaks, with the presumption that understanding these risk factors would illuminate the most effective sites of prevention for nosocomial infection.

While these events (and their historiography) are almost without exception set in the United States and Britain, their effect was to establish a set of standardized protocols that quickly became globalized. The International Nosocomial Infection Control Consortium (INICC) founded in Argentina in 1998 was explicitly modeled on and used the standardized forms developed in the setting of the American NNIS. In fact, we see the rough outline of this timeline echoed in the unfolding of events and practices at AUBMC, an institution with close professional and administrative ties to the US. Written for the AUB’s Board of Directors in New York, the Annual Reports of the hospital administrators after 1950 give a candid look into the importation of therapeutic modernity twinned with meticulous hygiene as part of the “liberalizing influences” of AUB’s mission in “an important area of the world which is awakening to new life,” as its Acting President Constantine Zurayk put it in the opening pages of the President’s Annual Report to the Board of Trustees for 1952–53 (AUB, 1953, pp. 4, 17). Hobart Reimann, a US virologist stationed at AUB, spoke on “Abuse of Antibiotics” to the Beirut Lebanese-French Medical Conference in 1953, a theme recurrent in the curriculum of the newly established Public Health School (Reimann, 1953).

In 1953, the newly appointed hospital administrator, Gilbert Nee, complained that “the housekeeping department is one of the weakest links in the Hospital” (Faculty of Medicine, AUB, 1954, p. 156). Steps were taken to replace the “simple but antiquated” local housekeeping practices using a straw broom and a piece of burlap with “new mops with wringers on ‘dollies,’ push brushes”; the ambition was that this would be “copied by other hospitals in this country [despite] many who feared that we could not change the century old habits and customs” (Faculty of Medicine, AUB, 1955, p. 185). *The Apothecary*, the School of Pharmacy’s yearbook, concertedly advertised antibiotics with “broad spectrum synergism” such as Pfizer’s Sigmamycin as a potential remedy for resistant staphylococcal infections (School of Pharmacy, AUB, 1957, p. 33) showing the global reach of combined therapy approaches to resistance detailed by Scott Podolsky in the American context (2015). Ascertainment that the principles of responsible antibiotic use and hygienic modernity had been satisfactorily implemented were no doubt part of the hospital’s accreditation by the American Joint Commission of Accreditation of Hospitals in 1955.

The Department of Bacteriology and Virology reported in 1959 that:

Repeated efforts were made to help the members of the faculty, staff and students realize the dangers of hospital acquired infections. Exhibits, movies, talks, distribution of mimeographed material, epidemiologic surveys and bacteriologic studies were used to this end. Methods were suggested to minimize the incidence of cross-infections in the hospital. (Faculty of Medicine, AUB, 1959, p. 109)

Hospital infection rate reports begin in the 1960s, with the Department of Surgery initiating post-operative infection rate reports in 1962. Routine clinical bacteriological screening for *Staphylococcus* infections began, and an outbreak of “hospital sepsis” in 1964 was written up by Professor of Bacteriology-Virology Robert Matossian in the *Lebanese Medical Journal*. Appointed in 1958, Matossian’s work shows that bacteriological work on the species and strains responsible for outbreaks had become part of hospital practice. He drew on British and U.S. pamphlets to contextualize the local situation in the hospital, including tracking nasal carriage of *Staphylococcus* by hospital personnel and studying linens as a vector of in-hospital transmission (Matossian, 1964).

The phage typing of the 1960s gave way to a new phase of research into antimicrobial resistant nosocomial infections in the early 1970s, with a focus on *Pseudomonas aeruginosa*. Serotyping and antibiotic susceptibility testing were used to track patterns of hospital outbreaks of *Pseudomonas*, with a focus on catheters, inhalation therapy and operating theaters as spaces of transmission within the hospital.^2^ For many years the Bacteriology-Virology Department, which was renamed Microbiology in 1977, doubled as the infection control body for the hospital, with Marwan Uwaydah taking a lead in the scientific study of hospital spread, and championing its importance. A student thesis by Sulafa Al-Dandan in 1990 noted that each decade seemed to come with its own group of hospital pathogens at AUH—*Staphylococcus* in the 1960s, MRSA and resistant Enterobacterales of various kinds in the 1970s, and *Acinetobacter* and *Pseudomonas* beginning to afflict the injured in the 1980s (Al-Dandan, 1990). Doctors, nurses, and microbiologists associated with the hospital went for extended research stays, mostly in the United States, and brought back styles of research inquiry, record-keeping, and even disinfectant choices. For example, the hospital began to purchase the phenolic disinfectant O’Syl, manufactured in the United States by Lehn and Fink (the makers of Lysol), to keep up with the shifting challenges presented by these outbreaks.

Although terse and highly bureaucratic, these records nonetheless gives lie to the idea that this was simply an adoption of the science, technology and clinical practice developed in the United States when facing the inevitable global nature of resistant infectious disease outbreaks under similarly globalized regimes of antibiotics and disinfectants. The yearly narrative from the hospital laboratories, housekeeping, and medical records division abruptly flickers out in the 1956–57 report as the hospital prepares for an expected disaster from the Suez crisis and the hospital director decamped to the United States. American troops landed in Beirut in July of 1958, called in to support the government of Camille Chamoun, and promptly come down in high numbers with dysentery, drawing on AUH resources such as laundry services. Only a year later, these same laundry services became the focus within the hospital of worries, with a physical renovation to quarantine soiled linen from clean, and a new testing regime focused on laundry’s role in hospital-acquired *Staphylococcus aureus* infections. The Nursing Service reported:

The past year is difficult to evaluate. A year ago we were undergoing a political crisis, and no report would be complete without reference to it. People went to the mountains and were frequently unable to return to the city. The hospital prepared to cope with casualties…wards were cleared and the unoccupied beds were used for our hospital staff as well as other employees of the University who could not commute because of road blocks and barricades. In the beginning there were a limited number of permits granted to employees for use during the hours of curfew and for a time the ambulance was used to pick up night staff and other key personnel (Faculty of Medicine, AUB, 1959, p. 247).

In addition, local data was considered very important in judging how to incorporate international standards to the immediate setting. Matossian worked tirelessly to understand the specificity of infectious disease in the country; he went on to publish a series of landmark books financially supported by AUB, such as *Infectious Diseases of Lebanon: Past, Present and Future* (1983). There is clear recognition in Lebanon of the specificity of the national context when it comes to the medical system (El-Jardali et al., 2011). Nonetheless, as social scientists Alex Broom and Assa Doron have put it, most orientations toward the global distribution of problems of AMR in the global health and the surveillance world “rely heavily on the notion of an objective material reality shared across contexts,” obscuring the physical specificity of non-shared socio-political relations salient in any given ecology of care, and overemphasizing behavioral adherence (or lack thereof) to international guidelines such as those around antibiotic use or hygiene (2022, p. 2). Following their call to “contaminate the imaginary of the clinic, so often treated as discrete and open to targeted intervention,” (2022, p. 2), we now turn away from the standard historiography in order to think about how the history of infection control and the identification of possible risk factors for resistant outbreaks departs in key aspects from the standard Euro-American story given above, when it is told as a social and environmental account in Lebanon.

While this list of departures from the standard historiography could be very long indeed, we highlight a few key points that emerged in our study of AUBMC’s institutional records and interviews of practitioners about their on-the-ground experiences of managing or studying AMR or environmental science in Lebanon after 1970. While these categories of war, patient transfer, and the place of state-level institutions are rather arbitrary and overlap to a certain degree—for example, issues of patient transfer are exacerbated by conditions of armed conflict and shaped by sectarianism—nonetheless, examining each in turn pulls out separate insights.

## War

Armed conflict looms large in the history of contemporary Lebanon and therefore must be included in any assessment of the social and environmental microbiology of the clinic in this setting. It may seem an obvious point that the exigencies of rendering care in hospitals under wartime conditions differ profoundly from the protocols and study designs around hospital hygiene after World War II in the UK and the US that were largely developed in conditions of relative peace (with the exception of military medical literature concerning the Korean and Vietnam wars). In Lebanon, periods of armed conflict are arguably *the* defining feature of AMR at AUBMC. Nonetheless, “war” is not a self-explanatory universal category, and its form was at this historical moment undergoing transformation. For example, Lebanon saw the escalation of novel forms of urbanized warfare. Conflicts in Beirut, Ashrafiye, Zahle, the Tel El-Zaatar Palestinian camp, Sidon and Tyre are listed in *Military Operations on Urbanized Terrain* as exemplars that “illustrate the trends, dominant factors, and principles of combat in urbanized areas” (US Marine Corps, 1998, p. 19).

Strategic destruction of city infrastructure, for example, was to be factored in in this new mode of war in cities:

Artillery was most effective in the interdiction of supplies, enemy evacuation, movement of reinforcements in the enemy’s rear (outside the city), and for indiscriminate physical and psychological harassment of the enemy. Artillery used as an indirect-fire siege weapon, as was done at Ashrafiyeh and Zahle, proved ineffective. Artillery can also cause problems for the attacker. The rubble resulting from indirect artillery fires can create considerable obstacles for the attacker while providing the defender with obstacles, materials, cover, and concealment.

The report further observes that aviation was central to the distribution of munitions into the conflict and the passage of the wounded away from it: “the IDF [Israeli Defense Forces] in Beirut II employed bombing by fixed-wing aircraft using cluster bomb units, “smart” bombs, phosphorous, and other munitions. Attack helicopters operated on the outskirts of the built-up area with impunity, and medical evacuation (MEDEVAC) proceeded swiftly and efficiently using helicopter support." (US Marine Corps, 1998, p. 26).

The social and environmental milieu of war as a specific driver of AMR in the twentieth century has not been fully conceptualized, and thus has gone under-investigated in terms of research (Fayad et al., 2023). Understanding the socio-material contours of a modern hospital in the middle of armed conflict can lend considerable insight into the specificity of the intersecting selective pressures that have shaped contemporary AMR. Importantly, these are not places of complete anarchy. The *Journal of the Arab Research Center for Injuries* in 1979 refers to the “organized chaos” by which the injured made their way to the hospital, by which blood supplies were reorganized and how, as we will see, the hospital itself reorganizes. These are spaces of making-do, in which new patterns of action are instituted to cope with the material challenges of the day.

Let us turn back to the hospital annual reports, which from the records of the 1958 crisis onward indicate important transformations to the clinical environment. A long list of violent events impacts the hospital’s operations—the so-called “Six-day war” or Third Arab–Israeli War of 1967, the bombing of Palestinian Liberation Organization (PLO) bases by Israel in 1972, and most profoundly, the Lebanese civil war, beginning in 1975. Here we focus on the 1975–1976 and 1976–77 reports. While there is no lack of historical work on this period’s dramatic events, they have not previously been considered in terms of microbial history (Fisk, 2001; Haugbolle, 2010; Traboulsi, 2012).

In these records we hear from the Dean of the Medical Faculty an account of the year’s events, but also from individual contributing units, such as Nursing, Housekeeping, and Bacteriology-Virology. As with every other sector of Lebanese society, the hospital was profoundly affected by the war that broke out in April of 1975: “the hospital overnight became a field-hospital,” receiving a constant stream of war casualties (Faculty of Medicine, AUB, 1976, p. 107). In 1975–1976 approximately 4666 wounded were treated, with 1207 of those admitted to the Hospital beyond treatment in the emergency room (Faculty of Medicine, AUB, 1976, p. 7); by the end of the next reporting year that estimate had climbed to 10,000 wounded receiving treatment (Faculty of Medicine, AUB, 1977, p. 13).^3^

It was not just the war wounded who were entering the hospital. “The stream of casualties who presented to the Emergency Room in large numbers and associated mainly with casualties among the fighting men (as well as frequently with civilian casualties also) were invariably accompanied by large numbers of armed men who insisted upon admission frequently at gun point” (Faculty of Medicine, AUB, 1976, p. 110). When power in Beirut shifted to one dominant player or another, the “controlling party, authorized by the Hospital administration to station guards at entrances, [required] armed persons entering the Hospital to deposit their weapons at control desks.” (Faculty of Medicine, AUB, 1977, p. 9). At points, “almost all the available beds were occupied by casualties” (Faculty of Medicine, AUB, 1976, p. 107).

As Richard Saliba, General Secretary of the Arab Research Center on Injuries reflected in the center’s journal, “To enter healthcare from the door of injuries is to enter through the backdoor—from the door of the Emergency Room, the ‘hot’ door. To reach the surgeon is the last resort, for we need, before reaching the surgeon, the citizen who aids himself first, and aids his brother citizen, and then the specialized medical aid, the skilled ambulance driver, and the field surgeon, before reaching the surgeon at the hospital” (1979).^4^ The dead also came, into the morgue. Concurrent with this influx was a severe drop in hospital employees able or willing to come to work, including janitorial, nursing, and medical faculty staff; the hospital itself was damaged by shelling in June 1976.

Significantly, the hospital faced severe infrastructural difficulties, notably in the supply of water. “Very serious problems arose during the summer and fall [of 1976]. During the fighting at Tel el Zaatar, water supplied to Ras Beirut was restricted, and at times completely cut off. Twice the AUH had only a four-hour supply.” (Faculty of Medicine, AUB, 1977, p. 8). Because of this restriction in supply to the neighborhood in which AUB is located, “Three wells on the lower campus were heavily pumped and the water soon became brackish. The mineral content made the water unpalatable and also damaged laundry equipment” (Faculty of Medicine, AUB, 1977, p. 9). Because of the intermittency of electricity from the municipal supply, the hospital often had to rely on generators. “Air cooling in the Hospital was discontinued because of the lack of water for the condensing towers as well as the shortage of electricity. Although air circulation to the subterranean areas was maintained, the Operating and Shock-Trauma Recovery Rooms became unbearably hot and several personnel fainted during prolonged procedures” (Faculty of Medicine, AUB, 1977, p. 9). The heat was enough to damage the x-ray machines and film.

In short, the hospital went through a protracted period of receiving wounded fighters and civilians in large numbers while nursing and medical staff shrunk by half or more. The finance section of the Annual Reports laments how the “catchment” area for the hospital became “narrowed” by the conflicts, only drawing those near the hospital, who often bring less income for the hospital. Elective procedures were discontinued and the challenging conditions were met with constant workarounds. One nurse recalls significant medical aid arriving to AUH, including disposable sterile supplies, and that the hospital’s Central Sterile Supply Unit remained operational, sterilizing water for burn patients. The hospital was relatively well off in terms of supplies provided by the US Federal Foreign Emergencies Fund, even while intermittent shortages of medications including antibiotics were noted in these years. An American nurse working at the hospital at this time remembers directly engaging with the warring parties in Beirut when drugs ran short, for example, “if they didn't have antibiotics, the surgeon will tell them, can you go get it from somewhere? They will get it, somehow they will get it. Somehow, we didn’t feel that it was a chaos, but an organized chaos by political parties” (Interview, 2022). At the same time, water, ventilation and cooling were frequently interrupted or not available.

In these records we see explicitly how “the environment” of war enters the hospital by many material entry points and follows a logic of “organized chaos” in which hygiene practices change in concert with many physical and biological factors. Such settings in which wartime conditions are a primary feature have been viewed as anarchic failures of rational modernity, not a source of insight into human-microbial coevolution. Yet what we see from this brief sampling of the records of the time is a reorganization of space, patients, outside visitors, the bodies of the dead, and where and how long they stay in different locations. Injuries were specific to the changing conditions of urban warfare, with the character of munitions and debris entering the hospital literally in the wounds to be treated as well as on the clothing and skin of the people moving through it. These changes intersect in space and time with environmental conditions of dust, water, ventilation and temperature. What we shall discuss at greater length below in considering the implications of the empirical characterization of such hospital environments is that these conditions presage a strikingly distinctive trajectory for AMR in hospitals—both in terms of the bacteria central to nosocomial infections and the reshaping of the hospital to contain these infections.

The cataloging of “organized chaos” given here provides a positive empirical frame in which to query how space and selective pressures driving antimicrobial resistance and spread are ordered in settings of social conflict. It is often assumed that under these conditions, hygiene becomes entirely impossible, and this by itself seems to tell you everything you need to know about the increased likelihood of bacterial infections, erratic antimicrobial treatment, and nosocomial spread. In other words, war is seen a condition in which all the *already known* risk factors for AMR development and spread are dialed up to maximum, thus verifying the significance of these risk factors identified elsewhere. What we draw from this short, detailed history of the hospital as an environment under the conditions of war is that there is still much to find out about the specificity of armed conflict as a driver of AMR. Certainly, these were not the prevailing concerns in Western hospitals in the period of establishment of research into the drivers of AMR, focused on antibiotic use and the clinical setting in peacetime; but they are part of the many worlds making up our one world.

## Patient transfers

Patient transfers came up repeatedly in our interviews with clinical microbiologists, AMR researchers, and hospital infection control personnel both in terms of discussing the history of the emergence of resistant nosocomial outbreaks as a focus of research energy and hospital response, and as part of the explanation of the ongoing evolution of the problem in Lebanon. Obviously, there is an overlap here with the discussion above of war as a factor; many interlocutors believed the origins of what became a recurring problem came into their hospitals in the bodies of patients wounded in armed conflict, either from Lebanon or from the region: “regarding *Acinetobacter*, it started also with a transfer of a patient from Iraq who came for his infected wounds and it started like that and then we couldn’t get rid of it until we took very drastic measures in the ICU” (Infectious disease specialist, 2021). Speaking about the early 2000s, a researcher noted that, “initially, we were seeing that patients infected with *Acinetobacter* mostly in those who came to us transferred from other hospitals and particularly among the wounded patients, you know, those who had been hospitalized, cared for in different hospitals, had chronic wounds, etc. But, within very few years…the organism became the most common cause of our nosocomial, not only respiratory tract infections, but also bloodstream infections in patients who had central catheters” (Infectious disease specialist, 2021).

The “drastic measures” mentioned above included outright closure of the ICU for a few weeks to disinfect and remodel. Yet a hospital cannot control the political geography of the country it is in, nor the social ties in which bacteria are as situated as humans are.

And then we had patients transferred from other hospitals. So, what happened is many other hospitals in Lebanon started dealing with the same struggle and so whenever there were transfers of patients, they will bring in a new strain, if you wish, but also a multidrug resistant strain. And then, you know, the story repeats itself. And at many points, we had to agree that we will refuse transfers from other facilities to the ICU because it became really very costly….And so we had to say, you know, we're not taking any more transfers, regardless, even if it's life-saving, etc. And so we'll do that, control and then, you know, someone will come, VIP patient, you know, affiliated with someone important in the country, and so that's why it was really a struggle and every time we put the plan, it would work for a few months, and then we had to deal with it again (Infectious disease specialist, 2021).

As political scientist Melani Cammett has noted, the establishment of more and less formal social welfare programs by Lebanon’s religious communities after “the partial and, for some programs, total breakdown of public social welfare institutions” during the Civil War (1975–1990) meant that control of access to medical services or the money to pay for them became a feature of personal or partisan ties, particularly for the poor (Cammett, 2015). These social ties are not a feature of the state or of any one private institution such as a hospital, but nonetheless shape the routes by which patients might move within a network of institutions in this particular place.

More recently, other multidrug resistant Gram-negative pathogens have been more of a problem than *Acinetobacter*, but the importance of the political and therapeutic geography of the country has become even more salient as economic crisis deepens (Dewachi et al., 2014). For example, in discussing the case of a soldier who was involved in a car accident in Beqaa and developed an infection while hospitalized and then was transferred to Beirut, this clinical microbiologist was describing “the way Lebanon is geographically distributed” as a driving factor for the spread of AMR. What he meant by this, he explained, was:

At the peripheries, at the borders of the country, you have poorer community, you have poorer hospitals, and then they start doing these interesting treatments, shall we call them, for patients and they start pumping them with antibiotics on the way into the hospital and then we get these patients transferred from these peripheral hospitals down to our hospitals in the cities and then you start seeing that the infection they have has gained resistance because of the stay in the hospital and not because the infection they have was resistant from the start… [one] patient became septic and he had bacteremia and he was about to lose his life because of the way he was being treated for the infection. There was no antibiogram done, there was even no culture done and they didn’t know what the bacterium was until we started working at AUB on it. Unfortunately, this is the case in the majority of the governmental hospitals that are found on the peripheries of the country, the hospitals in the cities they do have clinical microbiology, but the other ones, they barely have any. This is one of the reasons why we have AMR spreading (Clinical microbiologist, 2020).

This account aligns with a study published in 2019 that attempted to synthesize an overall picture of antimicrobial susceptibility data for Lebanon by compiling reports from individual hospitals; out of 152 hospitals (120 public, 32 private), 18 had the resources and procedures in place to generate yearly antibiogram reports (Moghnieh et al., 2019). An unevenness between institutions at both the level of treatment and the role of laboratory testing in that treatment is thus a fundamental condition shaping any transfer of an individual patient from one hospital to another.

Researchers working with data derived from Western hospital systems have emphasized the study of the network connecting different institutions—“hospitals cannot be viewed as individual units but rather should be viewed as connected elements of larger modular networks” (Donker et al., 2012, p. 1). Other studies consider the interior of ambulances as one spatial context connecting the different nodes in the network (Wepler et al., 2015); and the determination of inter- and intra-hospital transfer as an independent risk factor for nosocomial infection (Eveillard et al., 2001). Anywhere in the world, the transfer of patients between hospitals is an obvious potential mode of transmission of bacteria between hospitals; likewise, it is true anywhere that moving highly vulnerable ill and injured patients into a new setting or through the various spaces and conveyances on the way to a new setting can elevate the likelihood of that patient contracting a hospital-acquired infection in and of itself: that is to say, movement between institutions is a risk to patients, also anywhere in the world. Protocols for screening incoming transferred patients or limiting transfers would be readily seen as a principle at work in any hospital concerned with controlling nosocomial outbreaks, no matter where it is.

Nevertheless, in our efforts to track the history of AMR in Lebanon and in particular the historical unfolding of how the nosocomial pathogen *Acinetobacter* came to be established as a major medical problem in the 2000s, the factor of hospital transfer suggests that a suite of other factors should be considered *in any of these settings*. The situation in Lebanon points to the fact that any network of hospitals is not an undifferentiated space in which only the degree of connectedness matters, but a socioeconomically and politically stratified topology with centers and peripheries. Not all patients are equally likely to move between nodes; the different institutions may have very different (or no) capacities for screening patients and for determining antibiotic susceptibility profiles of pathogens; there may or may not be enough staff to perform the surveillance and isolation protocols that are officially in place.

While the historical formation of the political situation in Lebanon is distinctive and has many particularities that our interlocutors pointed to, we are not just arguing that Lebanon is special and universal protocols don’t apply. Rather, many urban centers in the world experience their own intersections of frequent gun violence and socioeconomic striation of both medical resources and environmental conditions. Therefore, it may well be that the principles one could develop by studying AMR in Lebanon could be productively adapted back to better understand the role of socioeconomic disparities, social ties, or religion in shaping patient transfer as a factor in AMR in Chicago or Paris or anywhere else. The assumption of the universality of biology and the failure to recognize social interaction and political environments as a feature of that biology is, paradoxically, a widely shared blind spot.

## The missing meso-level

Thus far we have mostly spoken in terms of global or international forces and local conditions, and their interaction. Yet one factor that repeatedly emerged in our interviews was that there is an important “meso” level intervening between the micro-history of any one institution or moment, and macro-histories of developments in global health. One microbiologist commented on a feeling of futility about maintaining strict control over the very local environment in these terms:

I mean our infection control is, for example at AUB, is very, very strict, you have other places that have infection control that are also quite strict and they're working quite well. The issue is that we don't have a national policy towards the reduction of AMR or we don’t have a national policy towards tackling antimicrobial resistance, and this is where the problem lies. Things are changing over time, yes, but these are all individual effort, or individual, what do you call it, preference, per se, where a hospital decides like I'm gonna start applying infection control program, I'm gonna start cutting down on antibiotic use, I'm gonna start cutting down prophylaxis antibiotics and start doing a little bit more screening and all of that, and that actually works, but the lack of a national effort to do this is what causes a lot more, how do you say, it's still what's causing problems to happen (Clinical microbiologist, 2020).

Because of the importance of surveillance, sample collection, diagnostic capacity, and coordination of communication and action (Maki & Zervos, 2021), this distinctive piece of the story of AMR in Lebanon could be called “the missing meso-level,” specific to the history of state formation and the place of science and medicine therein. Lebanon has been home to strong and established academic institutions dating back to the Ottoman Empire in the second half of the nineteenth century, the American University of Beirut (AUB, previously the Syrian Protestant College) and Université St-Joseph (USJ). The modern state of Lebanon came into being on the heels of these academic institutions already occupying much of the scientific landscape in microbiology, both locally and regionally. The historical specificity of the strength of these institutions vis-à-vis the State has meant that efforts to assert national-level microbiological governance have often faltered.

The project of creating a Lebanese Central Public Health Laboratory (CPHL) illustrates the challenges of building a meso-level national institution in a scientific landscape heavily influenced by existing strong academic institutions. The CPHL was legally established in 1956 (Lebanese Republic, 1956), two years before the crisis of 1958 that saw President Camille Chamoun met with revolt and be replaced by a third president of the Lebanese Republic, General Fouad Chehab, referred to by some as “Lebanon’s Last State-builder” (Hof, 2020). Chehab and his parliament had ambitious plans for the CPHL, geared towards prevention through surveillance of infectious diseases including polio, malaria and venereal disease. It provided, by legislative decree and at no cost to patients, preventive testing and vaccinations to the public “regardless of personal material conditions and nationality” (Lebanese Republic, 1959).^5^ Even then, the institution of the CPHL did not seem to have changed Lebanon’s established laboratory systems, which consisted, for the most part, of the private university laboratories of the American University of Beirut and Université Saint-Joseph, the pharmacological laboratories of which were legally recognized as “equivalent to the Central Public Health Laboratory” in 1960 (Lebanese Republic, 1960). The CPHL initially relied on the support and leadership of established researchers in these local academic institutions but its ability to carve out a significant mandate and recognition was hampered. The Civil War period of the 1980s saw the CPHL partially dismembered, operating as six Regional Public Health Laboratories across Lebanon’s governorates (Lebanese Republic, 1980), a step towards its total dismantlement in the 2000s at the height of the post-war reconstruction period.

As one of our interlocutors reflected, any hospital has its own institutional specificities that govern its patients, medical staff, and its spaces and flows, and yet the local practices instituted there bear the specific stamp of an absence of governance. The missing meso-level is an absence that profoundly shapes the local conditions of AMR, yet at the same time erodes the very knowability of AMR as a phenomenon one is in the midst of. Here we have a case of a failure to traverse scales from local to global, as the thing to be known disappears from systematic grasp at the intervening level. Such a phenomenon is interesting to consider from the point of Gabrielle Hecht’s call for the empirical use of “interscalar vehicles” that may be followed across scales of space and time (2018), or historian Deborah Coen’s call for attention to the very mobilization of scale itself by the scientists and institutions involved in the stakes of planetary problematics (2018). This is an interscalar vehicle that breaks down with the inability to traverse scale: one specificity of this situated history is its struggle to be situated in a context larger than itself but smaller than the entire world.

I believe that we did the most of what we can do. But if we had the support of the government, the Lebanese government, to gather all the information from other hospitals at that time, it would have been more enlightening for what we used to see in the hospital. Because we had an infection control program at that time, but other hospitals didn’t and antimicrobial treatments was not that regulated in other hospitals. It would have been nice to have it and to have the support of a national laboratory. We didn’t have it. We used to have a national laboratory in Lebanon, a national central laboratory in Lebanon, and it was closed…So, this national laboratory used to have aggregation, or assembly, or repository of all the isolates in Lebanon, especially water isolates, environmental isolates, were being studied. And it closed (Hospital Administrator, 2021).

The challenges posed by the absence of a CPHL were clearly exposed in a WHO report from 2016, which noted that “the national laboratory system is, to a large extent, disintegrated with each facility working alone”:

“Lebanon has high-quality laboratories that are able to conduct diagnostic tests for IHR (International Health Regulations) priority diseases. However, there is currently no central public health laboratory (CPHL), which is essential to improve preparedness and response, to set standards and regulations for laboratory quality and biosafety, and to ensure adequate monitoring and upgrades. A CPHL and a well-defined national laboratory network for both animal and human health would improve national coordination and ensure important reference functions to other laboratories.” (World Health Organization, 2016, pp. 30, 5)

Further, the report goes on to note that Lebanon’s Ministry of Public Health was not “fully linked with all hospitals and health facilities,” and even internally, the Ministry’s units were not “linked at a central level for real-time surveillance.” (World Health Organization, 2016, p. 34).

Despite many attempts to re-establish the CPHL, including promises of Ministers of Health and guarantees of funding by international actors including the World Health Organization, plans to reinstate it have, since then, routinely failed. To this day, Lebanon’s health and scientific laboratory infrastructures operate without a Central Public Health Laboratory, making Lebanon one of the few countries in the world to do so. Thus, Lebanon operates without national biorepository or biobanking capacities, with implications for sample collection, archiving, and scientific research, and forms an infrastructure deficit that has meant that, for the most part, historic isolates of samples from Lebanon are more likely to be found at the CDC in Atlanta, or at universities in Marseille, rather than in Beirut. While the patterns of movement of biological samples in many instances follows a colonial trajectory back toward metropoles in which there is money and infrastructure for collections, the loss or diaspora of samples as well as scientists in Lebanon is particularly extreme (Burton, 2022; Kirchhelle & Kirchhelle, 2022). This means there is a rather literal fragmentation of the matter of history, in the sense that one cannot just dig in a local freezer and see how clinical isolates in a single hospital have changed over time. This makes it difficult to systematically assess the biological and evolutionary consequences of a lack of systematicity. Paradoxically, a key characteristic of this “situated” history of microbes is the difficulty of anchoring the local situation within a set of nesting national and regional ones.

## Implications: theorizing one history, rethinking one health

What are the implications of these detailed descriptions of brackish well water and an “organized chaos” of tending to wounds sustained in armed conflict? In the absence of consistent collections of clinical samples from this time—a possibility abrogated by the very conditions described above—one cannot make direct causal claims about biology from historical documents alone. Nonetheless, we suggest that this “One History” approach—in which we have treated the hospital as possessing natural, social, and environmental history—is an important contributor to understanding the role of war in hospital hygiene, and to comprehending the hospital as an ecology of selective pressures and infection dynamics that is not other to environmental or One Health approaches but continuous with them.

One key implication of the stories we have unfolded above is that the historiography of hospital hygiene has for too long focused on AMR in high income countries, allowing a framing of hospital infections in LMICs as only a lack of resources or a failure to adhere to protocol. Instead, we have suggested that the specific conditions recounted may be understood as a “biology of history,” in which “human historical events and processes have materialized as biological events and processes and ecologies” (Landecker, 2016, p. 21). A limited historiography of AMR and hospital infection in which the topic of war fades after World War II cannot comprehend the biology of the history of Lebanon, which of course becomes consequential for hospital infection globally. In 2008, for instance, *A. baumannii* became the “A” in the acronym ESKAPE pathogens coined by Louis Rice to refer to the “coterie” of bacteria that in “hospitals in both the developed and developing world…cause the lion’s share of nosocomial infections” (2008, p. 1079).

While Europe and the United States were worrying about MRSA and struggling with ways to contain it in hospitals and patients—problems that dominated research and protocols around nosocomial infection in the 1980s—the American University Hospital in Beirut was by contrast encountering a very different trajectory.^6^ Hospital infection records, microbiological work, and personal recollections all point to the convergence of an influx of injuries from armed conflict with the emergence of the Gram-negative *Acinetobacter baumannii* as a nosocomial threat (Matar et al., 1992), which in turn transformed how infection control was approached in the hospital.

One of our interlocutors recalled that hospital AMR prior to the 1980s was “being monitored, singled out, but not feared.” Things changed only when “we started having a big outbreak and patients started to die having *Acinetobacter*, with *Acinetobacter*. This is when resources were given to us to increase the infection control team, the number of the infection control team members, to change, for example, the cleaning and disinfection solutions, to start new protocols, all these were backed up by administration after having the outbreak.” Both the initiating circumstances for these outbreaks—civil war—and the prevalence patterns for hospital pathogens were quite different from what was being experienced and studied in Europe and the United States at the time.

In fact, when this interviewee went to work for some months in the United States in the 1980s, she found that “they fear MRSA,” which was both a contrast and not particularly helpful in terms of the situation at home: “at that time we didn’t have MRSA, our patients were not colonized, we didn't have to do decolonization, etc. …Measures more prevalent in the US suited to MRSA were not enough to deal with *Acinetobacter*.” (Hospital Administrator, 2021) While MRSA was present in AUH and Lebanon more broadly in the 1970s—we can see it noted in both research and administrative records—the force of this recollection lies in the remembered professional contrast between the setting of an American hospital in America, and one in Beirut, and the inefficacy of emphasizing handwashing when faced with a pathogen such as *Acinetobacter*.

*Acinetobacter baumannii* was first detected as a nosocomial pathogen in the hospital in 1977, though other *Acinetobacter* species had been registered as benign skin colonizers of 125 outpatients “with no apparent dermatological disorders” in the thesis of Abdul-Razzak Taqi-Eddin in 1973 looking at non-fermenting Gram-negative species on skin samples (Taqi-Eddin, 1975).^7^ After another surge of violence in 1984, multiple serious outbreaks of *A. baumannii* in the hospital led to “a strain typing system to aid in infection control” and established the first system for epidemiologic typing of the pathogen (Matar et al., 1992). In this case, the world of the AUH literally traveled into what would become global protocol when Ghassan Matar took clinical samples from Lebanon to the CDC in Atlanta and worked with Frank Tenover to establish this system.

This system and the experience at AUH would become an important reference point for the subsequent prominence of this nosocomial pathogen once American soldiers injured in Iraq were deeply affected by multi-drug resistant infections recalcitrant to treatment, and it began to trouble the military hospitals in Germany and the United States that these soldiers moved through. Researchers at AUBMC continue today to be prominent contributors to the literature on infection prevention and control of carbapenem-resistant *A. baumannii* (Kanafani & Kanj, 2020; Kanafani et al., 2018; Rizk et al., 2022).

Obviously, we hardly need any further reminder that armed conflict is antithetical to human health and wellbeing. No one is going to stop waging war or passively sitting by while it happens elsewhere in the world because historical and biological evidence points to it as a specific driver of AMR. Yet this is a concern not just for war and AMR in Beirut or other places that have experienced or are experiencing armed conflict, but for microbiology around the globe. The point here is to assert a path forward for understanding hospital pathogens as socio-ecological events: the story unfolded here suggests that the emergence and spread of MDR pathogenic *A. baumannii* cannot be reduced to a failure of control measures focused on MRSA but must be fathomed within a natural and political and social history whose understanding requires more than a framework of lack.

## Conclusion: towards a situated biology for *bacteria*

Now, when we had opened our unit, we opened the COVID unit outside the hospital, along with the COVID ICU. So, it was a totally new ICU. And what happened was, in this new ICU, *because it has a story of its own*, it took us a very long time to get *Acinetobacter*. (Physician, 2021)

As clinicians and microbiologists increasingly move away from only examining AMR in clinical settings, a vibrant set of “One Health” approaches has fostered the search for and discovery of bacteria resistant to certain antibiotic thresholds far beyond the human body. The threat of AMR is then understood to lie everywhere, expanding the realm (and risk) of AMR to all ecosystems. Attention to antibiotics, mobile genetic elements carrying resistance determinants, and resistant bacteria in waterways, sewage effluents, animal husbandry, shrimp and lettuce, wildlife migration, and countless other environmental “hotspots” has increased dramatically in the literature over the last decade.

Paradoxically, this decontextualization of AMR from the primary locus of the (hospitalized) human body has had the unintended effect of rendering it miasmatic, just worse in the places where a universal set of risk factors is present. What we have endeavored to show here is that the biology of AMR is not just a feature of the interaction of humans, animals, plants, and abiotic factors such as wind and water in an Anthropocene ecosystem geologically and biotically shaped by human activity, but that the structure of human society in all the classic senses that sociologists, historians, and political scientists have understood—institutions, states, law, governance, sectarianism, and power—is woven into the biology of AMR in any given place (Fahmy, 2018). Each place, each ICU, as one of our interlocutors eloquently put it, “has a story of its own” in terms of how this socio-material history shapes the conditions of possibility for the pathogens that most worry humans.

As we have shown here, defining resistant bacteria and nosocomial infections according to primarily UK and US-based models and then disseminating these models globally is akin to a form of mobile scientific sovereignty, taking a scientific framework that mistakes its own social and institutional conditions for those of the entire world, and applying it in a decontextualized manner to other settings (Pandolfi, 2009). Reducing AMR to a decontextualized search for bacteria exhibiting resistance to antibiotics by lab-based methods will not be adequate to fathoming the material, social and environmental conditions in which bacteria become resistant, circulate, and ultimately cause infection. We have proposed, instead, to attend with careful empirical specificity to the materiality of history and the history of matter in the social and physical environments of the hospital, such that it can be thought of as a case of the interwoven situated biologies of humans and bacteria characterized by a “sociobiome” (Hinchliffe et al., 2018; Koch, 2011; Niewöhner & Lock, 2018). These interwoven situated (micro)biologies play out on different scales, from the macro (global, transnational, colonial), meso (national, societal) to the micro (often hospital-based) levels, and at different temporalities in the lifetimes of humans, microbes, and nations (Brives, 2021). Being attentive to all these levels, and comparing them across multiple different cases, is essential for efforts to grasp and attend to the issue of AMR.

## Footnotes

1 This article is based on empirical material collected through both oral history interviews and archival fieldwork. Interview-based fieldwork took place in two phases. Phase 1 interviews (between August 2020 and March 2021) included 14 scientists active in the area of environmental sciences and Phase 2 interviews (between September 2021 and January 2022) included 14 Lebanese scientists active in the area of hospital infection control. Interviewee selection was guided by scientists’ publication records in the areas of environmental and hospital microbiology and through selectively approaching participants who have decades-long experience in their respective fields. More specific details related to participants’ expertise are kept purposefully vague to protect their anonymity. Interviews were semi-structured and were held in-person, via video-conferencing, or both. All co-authors participated in conducting the interviews and in comparing interview transcripts for emerging themes. The interview-based phases of fieldwork received approval from the Université de Shérbrooke IRB. Archival research conducted by Anthony Rizk took place between January 2021 and September 2022 and was made possible through the archives of the Saab Medical Library of the American University of Beirut and the Medical Sciences Library of the Universite Saint-Joseph, both in Beirut. Main sources consulted included the *Revue Medicale de Moyen Orient* (1950–1954), *The Apothecary* (1951–1962), *the Lebanese Medical Journal* (1948–1999), the *Journal of the Arab Research Centre on Injuries* (1979), and the *Annual Report of the Faculty of Medicine and Medical Centre of the *American University of Beirut (1953–2013) and other select documents*.* Key information was extracted and mapped across a timeline for analysis. Sources are publicly available through the American University of Beirut’s University Libraries catalog (https://libcat.aub.edu.lb/). Student theses (1970–2015) were accessed through *AUB Scholarworks* (https://scholarworks.aub.edu.lb/).

2 Notably, this research was led by Nassim Nabbut of the AUB Faculty of Medicine’s Department of Microbiology, who later conducted what would today be termed a “One Health” study by serotyping *P. aeruginosa* from sheep and chicken in Saudi Arabia, concluding that the serotypes observed there matched those causing hospital outbreaks in the United States, Germany, and the one they had studied at AUB (Barbour et al., 1986); he also did extensive work on *Salmonella* in the Lebanese food supply.

3 Many of these were grievously wounded and in need of complex surgery, with “multiple injuries to the Chest, the Abdomen, the head as well as to the extremities, and many of these patients required multiple procedures and long hours of operating time to patch them up” (Faculty of Medicine, AUB, 1976, p. 109).

4 Translated from the original Arabic by Anthony Rizk.

5 This and other quotes from the relevant laws are translated from the original Arabic by Anthony Rizk.

6 MRSA was difficult to contain in part because hospital staff or patients themselves were often asymptomatic carriers who would unwittingly spread the bacteria to other people. The terminology for people in whom a bacterial population was established and ongoing (often in the nasal passages) was “colonized”.

7 At this time, Taqi-Eddin identifies *Herellea vaginicola* and *Mima polymorpha* in his 1975 thesis, conducted under the supervision of Marwan Uwaydah. Uwaydah and Taqi-Eddin would co-author, one year later, a taxonomical paper where they re-identify *Herellea vaginicola* as *Acinetobacter calcoaceticus* var. *anitratum* and *Mima polymorpha* as *Acinetobacter calcoaceticus* var. *lwoffi.* (Uwaydah & Taqi-Eddin, 1976).
